# Protective Effect of Tempol on Buthionine Sulfoximine-Induced Mitochondrial Impairment in Hippocampal Derived HT22 Cells

**DOI:** 10.1155/2016/5059043

**Published:** 2016-03-16

**Authors:** Ankita Salvi, Gaurav Patki, Eisha Khan, Mohammad Asghar, Samina Salim

**Affiliations:** Department of Pharmacological and Pharmaceutical Sciences, College of Pharmacy, University of Houston, Houston, TX 77204, USA

## Abstract

Using a simulated oxidative stress model of hippocampus-derived immortalized cell line (HT22), we report that prooxidant buthionine sulfoximine (BSO, 1 mM, 14 h), without adversely affecting cell viability or morphology, induced oxidative stress by inhibiting glutathione synthesis. BSO treatment also significantly reduced superoxide dismutase (SOD) activity (*p* < 0.05) and significantly lowered total antioxidant capacity (*p* < 0.001) in HT22 cells when compared to vehicle treated control cells. Antioxidant tempol, a piperidine nitroxide considered a SOD mimetic, reversed BSO-induced decline in SOD activity (*p* < 0.01) and also increased BSO-induced decline in total antioxidant capacity (*p* < 0.05). Interestingly, BSO treatment significantly reduced mitochondrial oxygen consumption (*p* < 0.05), decreased mitochondrial membrane potential (*p* < 0.05), and lowered ATP production (*p* < 0.05) when compared to vehicle treated control cells, collectively indicative of mitochondrial impairment. Antioxidant tempol treatment mitigated all three indicators of mitochondrial impairment. We postulate that BSO-induced oxidative stress in HT22 cells caused mitochondrial impairment, and tempol by increasing SOD activity and improving antioxidant capacity presumably protected the cells from BSO-induced mitochondrial impairment. In conclusion, present study provides an interesting simulation of oxidative stress in hippocampal cells, which will serve as an excellent model to study mitochondrial functions.

## 1. Introduction

Previously, we reported that chronic psychological stress-induced behavioral deficits in a variety of rat models were associated with an increase in oxidative stress in the brain, specifically the hippocampus [1–4]. Information regarding biochemical changes occurring within the hippocampus in response to oxidative stress is not well defined. Oxidative stress sensitive pathways seem to be involved in regulation of some of these behaviors [1], but how oxidative stress engages these pathways remains unclear. For instance, occurrence of hippocampal neuronal death following elevated oxidative stress was reported, but the pathway leading to cell death is uncertain [5]. We have focused our attention on one of the systems affected by oxidative stress, the mitochondria, which are abundantly present in the brain [6], making the brain highly vulnerable to oxidative stress [7].

Oxidative stress and the consecutive increase in levels of reactive oxygen species (ROS) within the mitochondria are reported to influence normal functioning of the electron transport chain (ETC), reducing mitochondrial oxygen consumption and consequent ATP production [8] and lowering mitochondrial membrane potential [9]. All of these factors presumably favor initiation of apoptosis [9] and disruption of mitochondrial fission and fusion protective machinery [10, 11]. Thus, oxidative stress seems to trigger impairment of mitochondrial function, mitochondrial degradation, and cell death [12, 13]. This is important, as maintenance of appropriate mitochondrial function is considered critical for regulation of stress response [14]. And hippocampal neurons have high bioenergetic demand and hence are quite susceptible to oxidative stress as well as to biochemical consequences resulting from occurrence of mitochondrial impairment [15].

This study was designed to address whether oxidative stress induces mitochondrial impairment by disrupting oxygen consumption, ATP synthesis, and membrane potential in a hippocampal derived mouse HT22 cell line. Basically, buthionine sulfoximine (BSO) was used as an oxidative stress-inducing agent. And the role of the antioxidant tempol, a piperidine nitroxide which functions as a superoxide dismutase (SOD) mimetic, in protecting against negative effects of BSO-induced oxidative stress on mitochondrial function also was examined.

## 2. Materials and Methods

### 2.1. Cell Culture

The immortalized mouse hippocampal (HT22) cell line was obtained from Dr. Dave Schubert from The Salk Institute, La Jolla. Cells were cultured in Dulbecco's modified Eagle's medium (DMEM, Invitrogen) containing 4.5 g of glucose/liter and supplemented with penicillin/streptomycin (50 units/mL), glutamate (2 mM), and 10% fetal bovine serum (Atlanta Biologicals, GA). Cells were incubated in a humidified chamber at 37°C with 10% CO_2_.

### 2.2. Experimental Scheme

HT22 cells were seeded into six-well cell culture plates and divided into four groups: control, tempol alone (3 mM in media), BSO alone (1 mM in media), and BSO + tempol. BSO was purchased from Sigma-Aldrich (St. Louis, MO) and tempol was purchased from Santa Cruz Biotechnology, Inc. (Dallas, TX). Dose of BSO (1 mM) was selected based on our previously published data [16]. Cells were treated with BSO or vehicle (DMEM media) when 60–70% are confluent. Tempol was added 10 h after addition of BSO. Cells were trypsinized at 14 h and used for various analyses. Thus, they are control (no treatment at 0 h, trypsinized at 14 h), BSO (1 mM BSO added at 0 h, trypsinized at 14 h), tempol (no treatment at 0 h, 3 mM tempol added at 10 h and trypsinized at 14 h), and BSO + tempol (1 mM BSO added at 0 h, 3 mM tempol added at 10 h and trypsinized at 14 h). All experiments were conducted at least 3-4 times.

### 2.3. Cell Viability and Morphology

Cell viability was determined using hemocytometer every 2 h following addition of BSO up to 20 h. Cell morphology was assessed at 0 h, 6 h, 14 h, and 18 h using a tissue culture microscope (model: CKX41, Olympus Corporation, Japan) with 10x magnification and 0.25 numerical aperture of the objective lens. The images were acquired using a color camera (Olympus CMOS camera, model number SC30) and a base-level acquisition software (Olympus Soft Imaging Solutions, Germany) was used.

### 2.4. 3-(4,5-Dimethylthiazol-2-yl)-2,5-diphenyltetrazolium Bromide (MTT) Assay

HT22 cells were seeded at 1.5 × 10^4^ in a 96-well plate in 200 *μ*L culture medium. The cells were incubated overnight in a humidified chamber at 37°C with 10% CO_2_. Cell count assay was conducted using commercially available MTT kit following the manufacturer's protocol (Molecular Probes, Grand Island, NY, Cat number V13154). The assay involved conversion of water soluble MTT (3-(4,5-dimethylthiazol-2-yl)-2,5-diphenyltetrazolium bromide) into an insoluble formazan. Concentration of solubilized formazan was used as a direct marker of viable cells and was determined by optical density at 570 nm. Absorbance at 570 nm for each group was plotted at 14 h and 18 h [17].

### 2.5. HT22 Cell Lysate

HT22 cells were harvested by trypsinization and cell pellet (approximately 7.5 × 10^5^ cells) was homogenized using RIPA buffer (Sigma-Aldrich Corp., St. Louis, MO, Cat number R0278). The homogenate was then centrifuged at 10,000 ×g for 15 minutes at 4°C and supernatant was collected. This supernatant was used for determining 8-isoprostane concentration, total antioxidant capacity, total glutathione, and SOD activity.

### 2.6. 8-Isoprostane Assay

HT22 cell lysate was used to measure levels of 8-isoprostane using an enzyme immunoassay (EIA) following manufacturer's protocol (Cayman Chemicals, Ann Arbor, MI, Cat number 516351).

### 2.7. Total Antioxidant Capacity

HT22 cell lysate was used to measure total antioxidant capacity following manufacturer's protocol (Cayman Chemicals, Ann Arbor, MI, Cat number 709001). The ability of the antioxidants in the sample to prevent oxidation of ABTS 2,2′-azino-di-[3-ethylbenzthiazoline sulphonate] to its oxidized form was compared with that of Trolox, a water soluble tocopherol analogue. Amount of oxidized ABTS produced was monitored by measuring absorbance at 750 nm and was quantified as millimolar Trolox equivalents.

### 2.8. Total Glutathione Assay

HT22 cell lysates were deproteinized according to manufacturer's protocol (Biovision, Milpitas, CA, Cat number K808-200). The deproteinized sample was then assayed to measure total glutathione (Cayman Chemicals, Ann Arbor, MI, Cat number 703002). Glutathione concentration was determined by measuring absorbance of TNB (5-thio-2-nitrobenzoic acid) at 405–414 nm.

### 2.9. Superoxide Dismutase (SOD) Assay

SOD activity was measured in HT22 cell lysates by following manufacturer's protocol (Cayman Chemicals, Ann Arbor, MI, Cat number 706002). SOD activity was determined by measuring amount of formazan dye produced by reading absorbance at 440 nm.

### 2.10. High Resolution Respirometry

HT22 cells were harvested by trypsinization and approximately 7.5 × 10^5^ cells were collected into complete medium, and respiration was measured at 37°C in a 2 mL chamber by high resolution respirometry using Oroboros Oxygraph series D and Datlab software (Oroboros Instruments, Innsbruck, Austria). Routine mitochondrial respiration, corrected for residual oxygen consumption due to oxidative side reactions, was measured in intact cells in complete medium. Datlab software was used to calculate oxygen consumption [18].

### 2.11. Mitochondrial Membrane Potential Measurement by JC-1

HT22 cells were seeded at 1.5 × 10^4^ in a 96-well plate in 200 *μ*L of culture medium. The cells were incubated overnight in humidified chamber at 37°C with 10% CO_2_. Mitochondrial membrane potential was determined using a kit based assay following the manufacturer's protocol (Cayman Chemicals, Ann Arbor, MI, Cat number 600880). The J-aggregates from JC-1 staining were measured with excitation and emission at 560 nm and 590 nm, respectively, with bandwidth of 10 nm. The monomers from JC-1 staining were measured with excitation and emission at 485 nm and 535 nm, respectively, with a bandwidth of 10 nm. The ratio of J-aggregate intensity (595 nm) to monomer intensity (535 nm) was plotted [19].

### 2.12. ATP Determination

HT22 cells were seeded at 3.5 × 10^4^ in 6-well plates in 2 mL culture medium. Sample preparation was performed as explained in [20] and ATP concentration was determined using a commercially available ATP Determination Kit (Molecular Probes, OR, Cat number A22066). The bioluminescence was measured at emission wavelength of 560 nm [21].

### 2.13. Western Blot Analysis

HT22 cell homogenates were prepared in RIPA buffer (Sigma-Aldrich Corp., St. Louis, MO) supplemented with protease inhibitor cocktail (Sigma-Aldrich Corp., St. Louis, MO). Protein concentration was determined in the lysates. The homogenates were next diluted with 4x Laemmli buffer and then subjected to SDS-polyacrylamide gel electrophoresis (PAGE) and western blotting was performed as previously published by us [1].

Antibody dilutions: glyoxalase-1 (GLO-1, ab81461, 1 : 1000) was purchased from Abcam (Cambridge, MA). Cu-Zn SOD (07-403, 1 : 1000) and Mn SOD (06-984, 1 : 1000) were purchased from Millipore (Temecula, CA). Glutathione-S-reductase-1 antibody (GSR-1, 1 : 200) was obtained from Dr. Iiris Hovatta (University of Helsinki, Finland). Following overnight incubation at 4°C, immunoreactive bands were detected by anti-rat, anti-rabbit, or anti-mouse horseradish peroxidase-conjugated secondary antibody (1 : 2000, Cell Signaling Technology, Danvers, MA). Chemiluminescence was detected by Alpha Ease FC 4.0 (Alpha Innotech Corp., San Leandro, CA) and densitometrically quantified using Fluorochem FC8800 software. All proteins were normalized to *β*-actin loading control (sc-47778, 1 : 1000, Santa Cruz Biotechnology, Santa Cruz, CA).

### 2.14. Statistical Analysis

All values were expressed as mean ± SEM. Significance was determined by Student's *t*-test (GraphPad Software, Inc., San Diego, CA). A value of *p* < 0.05 was considered significant.

## 3. Results

### 3.1. BSO Treatment Induced Oxidative Stress by Inhibiting Glutathione Synthesis and Reducing Antioxidant Capacity, without Disrupting Cell Morphology or Cell Viability

**Figure 1:**
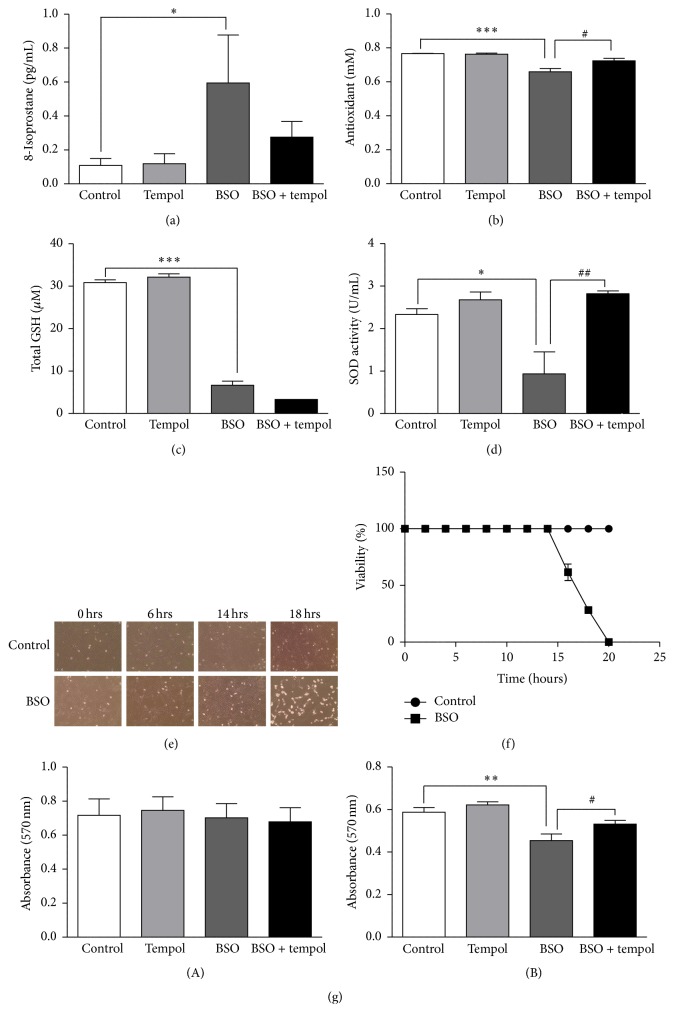
Analysis of BSO-induced oxidative stress, total glutathione levels, superoxide dismutase (SOD) activity, and cell morphology and viability in HT22 cells: representative graphs show (a) levels of 8-isoprostane (*n* = 5), (b) total antioxidant capacity (*n* = 8), (c) total glutathione concentration (*n* = 6), (d) SOD activity (*n* = 5), (e) representation of cell morphology (10x magnification) of control treated and BSO-treated HT22 cells at 0, 6, 14, and 18 h, (f) percentage of cell viability using hemocytometer (*n* = 3), and (g) cell viability using MTT assay following (A) 14 h and (B) 18 h of BSO treatment. Decrease in absorbance corresponds to lower cell viability (*n* = 8). ^*∗*^ *p* < 0.05, ^*∗∗*^ *p* < 0.01, and ^*∗∗∗*^ *p* < 0.001 significantly different from control; ^#^ *p* < 0.05 and ^##^ *p* < 0.01 significantly different from BSO-treated group. Bars represent means ± SEM.

14 h BSO treatment (i) increased 8-isoprostane levels (Figure 1(a), control: 0.10 ± 0.04 pg/mL, BSO: 0.59 ± 0.28 pg/mL, +490%, *p* < 0.05), (ii) reduced total antioxidant capacity (Figure 1(b), control: 0.76 ± 0.001 mM, BSO: 0.65 ± 0.01 mM, −14%, *p* < 0.001), (iii) decreased total glutathione (Figure 1(c), control: 30.86 ± 0.65 *μ*M, BSO: 6.63 ± 0.9 *μ*M, −78%, *p* < 0.001), and (iv) reduced SOD activity (Figure 1(d), control: 2.33 ± 0.13 U/mL, BSO: 0.93 ± 0.51 U/mL, −60%, *p* < 0.05). Tempol treatment alleviated 8-isoprostane levels, replenished the total antioxidant capacity, and reinstated SOD activity; however, it did not restore glutathione content to control levels.

Next, assessment of cell morphology indicated a progressive decline in cell count and normal morphology beyond 14 h BSO treatment when compared to control cells (Figure 1(e)). Hemocytometer count indicated 100% cell viability in BSO-treated groups up to 14 h of BSO treatment (Figure 1(f)). A progressive decrease in viability was observed beyond 14 h and complete cell death was observed at 20 h. Control groups were viable throughout the 20 h time period. Cell count also was verified using MTT assay. Absorbance at 570 nm did not change following 14 h BSO treatment (Figure 1(g)(A)). Thus, cell viability was not affected up to 14 h of BSO treatment. At 18 h, absorbance was reduced (Figure 1(g)(B), control: 0.58 ± 0.02, BSO: 0.45 ± 0.03, −22%, *p* < 0.01), indicating a decrease in cell viability. Tempol treatment restored cell viability.

### 3.2. BSO Treatment Induced Oxidative Stress by Suppressing the Level of Antioxidant Enzymes

**Figure 2:**
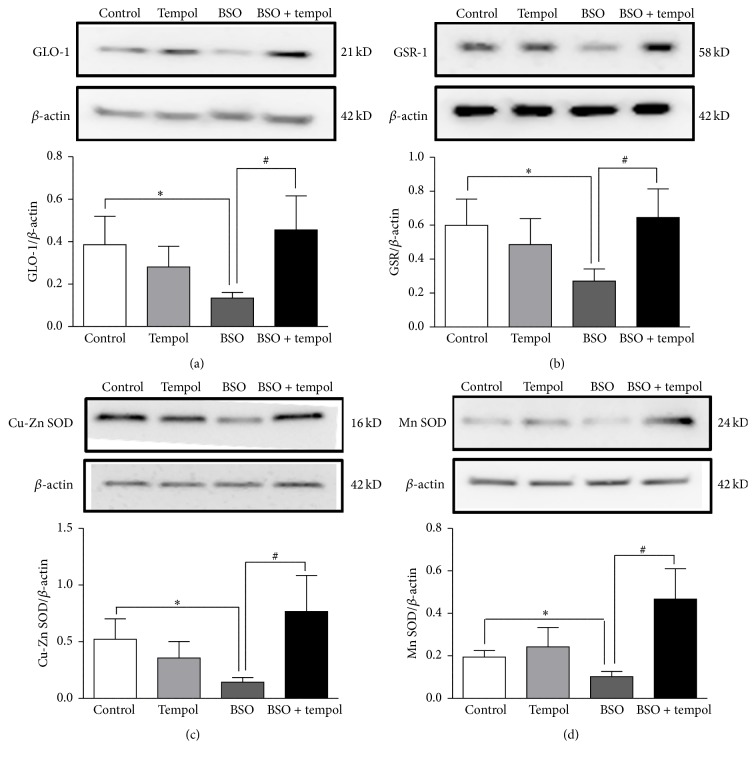
Analysis of antioxidant protein levels by western blotting. Protein levels of (a) GLO-1, (b) GSR-1, (c) Cu-Zn SOD, and (d) Mn SOD were determined by western blotting. Shown are representative blots and densitometric ratios of proteins normalized to *β*-actin, respectively; ^*∗*^significantly different from control; ^#^significantly different from BSO-treated group at *p* < 0.05. Bars represent means ± SEM, *n* = 5–9.

There was decrease in protein expression of (i) GLO-1 (Figure 2(a), control: 0.38 ± 0.13, BSO: 0.13 ± 0.02, −65%, *p* < 0.05), (ii) GSR-1 (Figure 2(b), control: 0.59 ± 0.15, BSO: 0.27 ± 0.07, −54%, *p* < 0.05), (iii) Cu-Zn SOD (Figure 2(c), control: 0.52 ± 0.18, BSO: 014 ± 0.04, −73%, *p* < 0.05), and (iv) Mn SOD (Figure 2(d), control: 0.19 ± 0.03, BSO: 0.10 ± 0.02, −47%, *p* < 0.05) following 14 h BSO treatment. Tempol treatment restored the expression of these proteins. All values are expressed as ratio of individual proteins normalized to *β*-actin.

### 3.3. BSO Treatment Reduced Mitochondrial Oxygen Consumption, Lowered Mitochondrial Membrane Potential, and Inhibited ATP Synthesis

**Figure 3:**
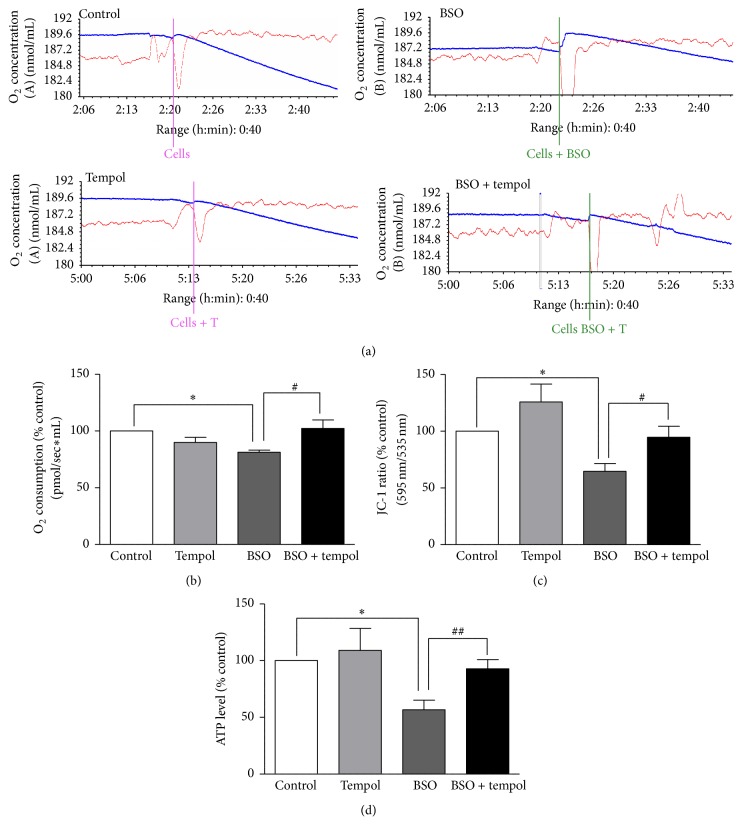
Analysis of mitochondrial oxygen consumption, mitochondrial membrane potential, and ATP levels in HT22 cells. (a) Representative oxygraphs showing respiratory activity of the HT22 cells. (b) The measurements were performed with 7.5 × 10^5^ cells per assay. The rate of oxygen consumption is shown in pMO_2_/mL and represented as % control (*n* = 4), (c) mitochondrial membrane potential data presented as a ratio of J-aggregates intensity (595 nm) to monomer intensity (535 nm). The JC-1 ratio was calculated as % control (*n* = 8). (d) ATP levels of the cells were evaluated using the luciferase assay system. Data is represented as % control (*n* = 8). ^*∗*^ *p* < 0.05 significantly different from control; ^#^ *p* < 0.05, ^##^ *p* < 0.01 significantly different from BSO. Bars represent means ± SEM.

BSO treatment (i) reduced mitochondrial oxygen consumption (Figures 3(a) and 3(b), control: 100%, BSO: 81.34 ± 1.8%, *p* < 0.05), (ii) lowered mitochondrial membrane potential (Figure 3(c), control: 100%, BSO: 64.64 ± 6.87%, *p* < 0.05), and (iii) decreased ATP synthesis (Figure 3(d), control: 100%, BSO: 56.71 ± 8.5%, *p* < 0.05). Tempol treatment restored the mitochondrial oxygen consumption, reestablished mitochondrial membrane potential, and restored ATP synthesis.

## 4. Discussion

Using a simulated HT22 model of oxidative stress, we have obtained exciting new information.* First*, the prooxidant role of BSO at a dose of 1 mM after 14 h treatment was established in this cell model, where significant oxidative stress was observed without disruption of cell number, cell morphology, or cell viability.* Second*, BSO is reported to generate oxidative stress by inhibiting glutathione synthesis [22]. Therefore, glutathione levels were evaluated and decreased total glutathione content was observed following 14 h BSO treatment, confirming the inhibitory action of BSO on glutathione synthesis. These observations were consistent with a previous study [23] in which 1 mM BSO treatment led to elevation of oxidative stress in the HT22 cell model without altering cell viability. Moreover, in a previous study, we had observed increase in oxidative stress following 1 h treatment of the same dose of BSO (1 mM) in CATH.a cells, a neuronal cell line derived from the locus coeruleus (LC) region of the brain. This suggests that the prooxidant activity of BSO is not limited to immortalized cell lines such as HT22 but has also been observed in other neuronal cell lines [16].

*Third*, increase in oxidative stress is believed to be associated with decrease in the levels of antioxidant enzymes as well as reduced activity of these enzymes particularly superoxide dismutase (SOD) [24]. Therefore, expression of endogenous antioxidant enzymes such as GLO-1, GSR-1, Cu-Zn SOD, and Mn SOD was measured following BSO treatment. Relevant to this, increased levels of oxidative stress marker 8-isoprostane following 14 h BSO treatment were associated with a significant decrease in total antioxidant capacity as well as reduced expression of SOD and other antioxidant enzymes. Thus, apart from a direct inhibitory action on glutathione, BSO indirectly suppressed endogenous antioxidant defense system.

SOD by scavenging free radicals such as superoxides is reported to mitigate generation of oxidative stress [25]. Antioxidant tempol is considered SOD mimetic [26]. Therefore, tempol's mitigating effects were tested in our cell culture model. Tempol treatment alleviated BSO-induced oxidative stress by restoring protein expression of SOD and other antioxidant enzymes. Tempol also increased both SOD activity and total antioxidant capacity when compared to BSO-treated cells. Though tempol's primary action is to scavenge superoxides [26], the resultant alleviation of oxidative stress presumably led to restoration in expression of endogenous antioxidant enzymes. This could be the probable explanation behind tempol's ability to restore total antioxidant capacity. Thus, we successfully simulated oxidative stress with BSO and tested tempol's antioxidant action in our cell culture model.

Next, we proceeded to investigate the effect of BSO on mitochondrial function. Generation of ROS within the mitochondria is believed to disrupt the electron transport chain (ETC), which leads to decreased mitochondrial oxygen consumption, hampering ATP production [8], and lowering mitochondrial membrane potential [9]. All of these events collectively contribute to mitochondrial dysfunction which could eventually compromise cellular integrity. In the present study, BSO treatment led to decrease in mitochondrial oxygen consumption, reduced mitochondrial membrane potential, and lowered ATP synthesis when compared to control cells. However, at this time point (14 h), BSO-induced oxidative stress was probably not enough to cause caspase-mediated apoptosis which is generally the subsequent step following mitochondrial dysfunction [9]. This could be the possible explanation for unaltered cell viability at 14 h. Interestingly, tempol treatment reversed BSO-induced mitochondrial dysfunction. This suggests that tempol, by enhancing SOD activity, improves the total antioxidant capacity of HT22 cells which protects the cells from oxidative stress-induced mitochondrial dysfunction.

Our overall hypothesis is that glutathione depletion with BSO leads to accumulation of ROS in the cells, specifically the mitochondria. ROS cause DNA oxidation, potentially introducing mutations in mitochondrial DNA which could cause disruption of ETC complexes such as complexes I and III [27]. ETC disruption leads to mitochondrial dysfunction. Tempol, by scavenging superoxides, brings down the level of ROS, thus preventing mitochondrial DNA damage and ETC disruption and hence protecting mitochondrial dysfunction from occurring. Our future studies would involve testing this hypothesis.

## 5. Conclusion

In conclusion, our results demonstrate that BSO by inhibiting glutathione synthesis generates excessive oxidative stress, consequently disrupting mitochondrial function and decreasing oxygen consumption. Less utilization of oxygen lowers ATP production causing energy depletion in cells. Also, BSO lowers mitochondrial membrane potential which most likely results in leaky membrane ultimately damaging cell integrity [9]. We also report that treatment with the antioxidant tempol alleviates oxidative stress and restores mitochondrial function. Collectively, our study suggests causal role of oxidative stress in causing mitochondrial impairment and also supports protective role of tempol against mitochondrial dysfunction. Finally, this study provides an excellent hippocampal derived cell culture model to study mitochondrial functions. This is particularly relevant considering high susceptibility of hippocampal neurons to oxidative stress, disruption of normal mitochondrial function, and stress responsiveness [15].

## Abbreviations

HT22: Immortalized mouse hippocampal cell line
BSO: Buthionine sulfoximine
ETC: Electron transport chain
SOD: Superoxide dismutase
ROS: Reactive oxygen species
GLO-1: Glyoxalase-1
GSR-1: Glutathione-S-reductase-1
DMEM: Dulbecco's modified Eagle's medium
MTT: 3-(4,5-Dimethylthiazol-2-yl)-2,5-diphenyltetrazolium bromide
EIA: Enzyme immunoassay
ABTS: 2,2′-Azino-di-[3-ethylbenzthiazoline sulphonate]
TNB: 5-Thio-2-nitrobenzoic acid.
